# Mental health interventions for adolescents with depression and anxiety in low- and middle-income countries: a systematic review of the evidence

**DOI:** 10.3389/fpsyg.2026.1809434

**Published:** 2026-10-19

**Authors:** Shuya Yin, Tian Tian, Wendy Auslander

**Affiliations:** 1 George Warren Brown School of Social Work, Washington University in St. Louis, St. Louis, MO, United States; 2 School of Social Practice,University of Auckland, Auckland, New Zealand

**Keywords:** adolescent, anxiety, depression, low and middle-income countries, mental health, systematic review

## Abstract

**Background:**

One in seven adolescents aged 10 to 19 worldwide suffers from mental disorders. Anxiety and depression disorders makeup about 40% of mental disorders. Adolescents in low and middle-income countries (LMICs) are particularly vulnerable due to limited access to evidence-supported mental health services. The current systematic review examined the strength of the evidence among interventions designed to reduce depression and anxiety symptoms among adolescents in LMICs, particularly comparing the effectiveness of school versus non-school-based interventions, as well as the training of the intervention providers (specialists versus non-specialists).

**Methods:**

Twenty-two studies were included after a systematic search and screening. The Risk-of-Bias (RoB) assessment and the Methodological Quality Rating Scale (MQRS) were used to assess methodological rigor. The certainty of the evidence for each study was evaluated by the Grading of Recommendations Assessment, Development and Evaluation (GRADE).

**Results:**

The search resulted in 22 trials with 2,336 participants across 11 countries. The findings indicated the potential effectiveness of non-specialist versus specialist-delivered interventions for depression with high certainty. The evidence was insufficient to determine whether school-based or non-school-based interventions were more effective for either depression or anxiety.

**Conclusion:**

Our findings suggest that future non-specialist-delivered interventions might be an important approach for reducing adolescents’ depression symptoms. More rigorous research is needed to determine whether this task-shifting approach is similarly effective for reducing anxiety, and whether particular implementation settings offer additional benefit.

**Systematic review registration:**

Publicly accessible website: https://www.crd.york.ac.uk/PROSPERO/view/CRD42024598603, and PROSPERO ID is CRD42024598603.

## Introduction

Adolescence is a critical developmental stage with a heightened risk of developing mental health disorders such as anxiety and depression (Suppiej et al., 2025). According to the World Health Organization, these mental health disorders are the leading causes of illness and disability among adolescents (World Health Organization, 2024b). The global prevalence of mental health problems among adolescents is substantial, with an estimated 35% experiencing depressive symptoms (Shorey et al., 2022) and 10% experiencing anxiety symptoms (Biswas et al., 2020; Shorey et al., 2022). Those youths exhibiting depression and anxiety symptoms faced increased risks of long-term mental health problems in adulthood, lower quality of life, and reduced academic achievement (Copeland et al., 2015; Colder et al., 2018). Mental health problems also have increased the economic burden to society. The State of the World’s Children Report 2021 estimated that the global cost of lost human capital due to adolescent mental health conditions was $451 billion annually and was expected to rise to $620 billion by 2040.

Adolescents in low- and middle-income countries (LMICs), where 90% of the world’s adolescents live (Rose-Clarke et al., 2024), may be particularly vulnerable due to financial constraints and limited access to evidence-supported mental health services. The prevalence of untreated severe mental disorders was over twice as high in LMICs (76–85%) compared to high-income countries (35–50%) (World Health Organization, 2021). Yet, the health systems in LMICs have not adequately responded to the burden of mental health issues in this population (Wakida et al., 2018). Several barriers have contributed to the low treatment rates, such as low mental-health literacy, stigma related to mental health problems, limited access to services due to inadequate training, and a shortage of mental health specialists (Mascayano et al., 2015; Mohammadzadeh et al., 2020; Zemestani et al., 2024). Thus, there is an urgent need to implement prevention and early intervention strategies in LMICs to alleviate adolescent depression and anxiety comorbidity and reduce social costs.

To address these challenges, the WHO published the Comprehensive Mental Health Action Plan 2013–2030 (World Health Organization, 2013), which highlighted the need for responsive mental health services and research in LMICs. In response, mental health interventions for adolescents in LMICs have rapidly increased over the past decade. Although there have been several systematic reviews that have examined the effectiveness of interventions targeting depression and anxiety among adolescents since the initial WHO report (2013), there are several knowledge gaps in the literature. First, most of the previous systematic reviews focused on universal interventions for the general population or treatment interventions for adolescents with a clinical diagnosis (e.g., Alozkan-Sever et al., 2023; Klasen and Crombag, 2013; Purgato et al., 2018; Uppendahl et al., 2020; Yatham et al., 2018). These reviews showed that psychological interventions had positive effects in reducing symptoms of depression and anxiety among adolescents in LMICs. However, few reviews have targeted interventions for participants with subclinical anxiety or depression, which include symptoms of anxiety or depression but do not meet the criteria for psychiatric disorders (Beesdo et al., 2009). To respond this gap, the current review focused on interventions targeted at high-risk adolescents who experienced subclinical anxiety and/or depression. Second, most systematic reviews rated the methodological quality and effectiveness of each study but did not assess the strength of the evidence. In this review, the strength of evidence of each study was assessed by examining outcome significance while taking into account methodological rigor.

Third, implementation challenges persist due to limited human and financial resources in LMICs. Under the severe shortage of mental health professionals, World Health Organization (2013) promoted expanding mental health services through a task-shifting approach in LMICs. Task-shifting refers to redistributing specific health tasks from highly trained specialists to non-specialist providers with targeted training. Galvin and Byansi’s (2020) review and Karyotaki et al.'s (2022) meta-analysis have found task-shifting for mental health service delivery to be a promising approach in LMICs contexts. Yet no systematic reviews have systematically compared the effectiveness of interventions by implementation site (school-based versus non-school-based) and training of service providers (specialists vs. non-specialists).

Therefore, in order to address these gaps, the current systematic review examined the strength of the evidence among interventions designed to reduce depression and anxiety among adolescents in LMICs, particularly comparing the effectiveness of school- versus non-school-based interventions, as well as the training of the intervention providers (specialists versus non-specialists). Specifically, this systematic review answered the following research questions: (1) What types of psychological interventions were used to reduce depression and anxiety among adolescents in LMICs? (2) What was the methodological quality and the risk of bias of these intervention studies? (3) Which types of interventions (i.e., school-based versus non-school; specialist versus non-specialist-provider) were most effective at reducing depression and anxiety, considering the studies’ methodological quality and risk of bias?

## Method

### Protocol and registration

This systematic review was conducted and reported in accordance with the PRISMA 2020 statement (Page et al., 2021) and the Synthesis Without Meta-analysis (SWiM) reporting guidelines (Campbell et al., 2020). The authors developed a search protocol, inclusion and exclusion criteria, and a data extraction table to document details throughout the review process. This review was registered on PROSPERO (ID: CRD42024598603).

### Search strategy

A systematic literature search was conducted on the following databases: Academic Search Complete (January 1, 2013 – July 31, 2024), APA PsycInfo (January 1, 2013 – July 31, 2024), Global Health (January 1, 2013 – July 31, 2024), MEDLINE (January 1, 2013 – July 31, 2024), SocINDEX with Full Text (January 1, 2013 – July 31, 2024). Search terms and databases were developed after consulting with a social science university-based librarian. Full search strategies for each database can be found in Table 1. The same search strategy was applied consistently across all databases. Gray literature was excluded from this review. After identifying references, Zotero and Excel were used to manage the literature and the screening process.

**Table 1:** Search terms used for systematic database searches.

Search strategy
Databases searched: Academic Search Complete, APA PsycInfo, Global Health, MEDLINE, SocINDEX with Full Text
Population: “adolescen*” OR “high school*” OR “middle school*” OR “middle childhood” OR “preadolescen*” OR “preteen*” OR “secondary school*” OR “teen*” OR “senior high student*” OR “youth*” OR “emerging adult*” AND
Country: “Algeria” OR “Ghana” OR “Micronesia” OR “Sao Tome and Principe” OR “Angola” OR “Haiti” OR “Mongolia” OR “Senegal” OR “Bangladesh” OR “Honduras” OR “Morocco” OR “Solomon Islands” OR “Benin” OR “India” OR “Myanmar” OR “Sri Lanka” OR “Bhutan” OR “Indonesia” OR “Nepal” OR “Tajikistan” OR “Bolivia” OR “Iran” OR “Nicaragua” OR “Tanzania” OR “Cambodia” OR “Ivory Coast” OR “Nigeria” OR “Timor-Leste” OR “Cameroon” OR “Kenya” OR “Pakistan” OR “Tunisia” OR “Cape Verde” OR “Kiribati” OR “Palestine” OR “Ukraine” OR “Comoros” OR “Kyrgyzstan” OR “Papua New Guinea” OR “Uzbekistan” OR “Djibouti” OR “Laos” OR “Philippines” OR “Vanuatu” OR “Egypt” OR “Lebanon” OR “Republic of the Congo” OR “Vietnam” OR “El Salvador” OR “Lesotho” OR “Samoa” OR “Zimbabwe” OR “Eswatini” OR “Mauritania” OR “Albania” OR “Cuba” OR “Kazakhstan” OR “Paraguay” OR “American Samoa” OR “Dominica” OR “Kosovo” OR “Peru” OR “Argentina” OR “Dominican Republic” OR “Libya” OR “Russia” OR “Armenia” OR “Ecuador” OR “Malaysia” OR “Saint Lucia” OR “Azerbaijan” OR “Equatorial Guinea” OR “Maldives” OR “Saint Vincent and the Grenadines” OR “Belarus” OR “Fiji” OR “Marshall Islands” OR “Serbia” OR “Belize” OR “Gabon” OR “Mauritius” OR “South Africa” OR “Bosnia and Herzegovina” OR “Georgia” OR “Mexico” OR “Suriname” OR “Botswana” OR “Grenada” OR “Moldova” OR “Thailand” OR “Brazil” OR “Guatemala” OR “Montenegro” OR “Tonga” OR “Bulgaria” OR “Guyana” OR “Namibia” OR “Turkey” OR “China” OR “Iraq” OR “North Macedonia” OR “Turkmenistan” OR “Colombia” OR “Jamaica” OR “Palau” OR “Tuvalu” OR “Costa Rica” OR “Jordan”) OR SU (“Algeria” OR “Ghana” OR “Micronesia” OR “Sao Tome and Principe” OR “Angola” OR “Haiti” OR “Mongolia” OR “Senegal” OR “Bangladesh” OR “Honduras” OR “Morocco” OR “Solomon Islands” OR “Benin” OR “India” OR “Myanmar” OR “Sri Lanka” OR “Bhutan” OR “Indonesia” OR “Nepal” OR “Tajikistan” OR “Bolivia” OR “Iran” OR “Nicaragua” OR “Tanzania” OR “Cambodia” OR “Ivory Coast” OR “Nigeria” OR “Timor-Leste” OR “Cameroon” OR “Kenya” OR “Pakistan” OR “Tunisia” OR “Cape Verde” OR “Kiribati” OR “Palestine” OR “Ukraine” OR “Comoros” OR “Kyrgyzstan” OR “Papua New Guinea” OR “Uzbekistan” OR “Djibouti” OR “Laos” OR “Philippines” OR “Vanuatu” OR “Egypt” OR “Lebanon” OR “Republic of the Congo” OR “Vietnam” OR “El Salvador” OR “Lesotho” OR “Samoa” OR “Zimbabwe” OR “Eswatini” OR “Mauritania” OR “Albania” OR “Cuba” OR “Kazakhstan” OR “Paraguay” OR “American Samoa” OR “Dominica” OR “Kosovo” OR “Peru” OR “Argentina” OR “Dominican Republic” OR “Libya” OR “Russia” OR “Armenia” OR “Ecuador” OR “Malaysia” OR “Saint Lucia” OR “Azerbaijan” OR “Equatorial Guinea” OR “Maldives” OR “Saint Vincent and the Grenadines” OR “Belarus” OR “Fiji” OR “Marshall Islands” OR “Serbia” OR “Belize” OR “Gabon” OR “Mauritius” OR “South Africa” OR “Bosnia and Herzegovina” OR “Georgia” OR “Mexico” OR “Suriname” OR “Botswana” OR “Grenada” OR “Moldova” OR “Thailand” OR “Brazil” OR “Guatemala” OR “Montenegro” OR “Tonga” OR “Bulgaria” OR “Guyana” OR “Namibia” OR “Turkey” OR “China” OR “Iraq” OR “North Macedonia” OR “Turkmenistan” OR “Colombia” OR “Jamaica” OR “Palau” OR “Tuvalu” OR “Costa Rica” OR “Jordan”) OR AB (“Algeria” OR “Ghana” OR “Micronesia” OR “Sao Tome and Principe” OR “Angola” OR “Haiti” OR “Mongolia” OR “Senegal” OR “Bangladesh” OR “Honduras” OR “Morocco” OR “Solomon Islands” OR “Benin” OR “India” OR “Myanmar” OR “Sri Lanka” OR “Bhutan” OR “Indonesia” OR “Nepal” OR “Tajikistan” OR “Bolivia” OR “Iran” OR “Nicaragua” OR “Tanzania” OR “Cambodia” OR “Ivory Coast” OR “Nigeria” OR “Timor-Leste” OR “Cameroon” OR “Kenya” OR “Pakistan” OR “Tunisia” OR “Cape Verde” OR “Kiribati” OR “Palestine” OR “Ukraine” OR “Comoros” OR “Kyrgyzstan” OR “Papua New Guinea” OR “Uzbekistan” OR “Djibouti” OR “Laos” OR “Philippines” OR “Vanuatu” OR “Egypt” OR “Lebanon” OR “Republic of the Congo” OR “Vietnam” OR “El Salvador” OR “Lesotho” OR “Samoa” OR “Zimbabwe” OR “Eswatini” OR “Mauritania” OR “Albania” OR “Cuba” OR “Kazakhstan” OR “Paraguay” OR “American Samoa” OR “Dominica” OR “Kosovo” OR “Peru” OR “Argentina” OR “Dominican Republic” OR “Libya” OR “Russia” OR “Armenia” OR “Ecuador” OR “Malaysia” OR “Saint Lucia” OR “Azerbaijan” OR “Equatorial Guinea” OR “Maldives” OR “Saint Vincent and the Grenadines” OR “Belarus” OR “Fiji” OR “Marshall Islands” OR “Serbia” OR “Belize” OR “Gabon” OR “Mauritius” OR “South Africa” OR “Bosnia and Herzegovina” OR “Georgia” OR “Mexico” OR “Suriname” OR “Botswana” OR “Grenada” OR “Moldova” OR “Thailand” OR “Brazil” OR “Guatemala” OR “Montenegro” OR “Tonga” OR “Bulgaria” OR “Guyana” OR “Namibia” OR “Turkey” OR “China” OR “Iraq” OR “North Macedonia” OR “Turkmenistan” OR “Colombia” OR “Jamaica” OR “Palau” OR “Tuvalu” OR “Costa Rica” OR “Jordan” OR “restricted resources countr*” OR “middle income countr*” OR “restricted resources countr*” OR “LMIC”) OR SU (“restricted resources countr*” OR “middle income countr*” OR “restricted resources countr*” OR “LMIC”) OR AB (“restricted resources countr*” OR “middle income countr*” OR “restricted resources countr*” OR “LMIC” AND
Intervention: “treatment*” OR “intervention*” OR “therap” OR “psychotherap” OR “counseling” OR “counselling” OR “training*” OR “psychoeducation” OR “promot*” OR “prevent*” OR “Program*” OR “Home visiting” OR “Support” OR “project*” OR “Initiative*” OR “group work” OR “groupwork” OR “supportgroup*” OR “support group*” OR “peer led support*” OR “task shifting” OR “postvention” OR “postintervention” OR “yoga” OR “meditation” OR “motivational interview*” OR psychosocial*” OR “mindful*” AND
Mental Health: “mental health” OR “mental illness” OR “mental disorder” OR “anxiety” OR “depression” OR “trauma*” OR “mental problem*” OR “mental disease*” OR “psychiatri*” AND
Comparison: “control* trial*” OR “quasi experiment*” OR “quasiexperiment*” OR “RCT” OR “randomi*” OR “arm*”

The search terms used free-text keywords with truncation (e.g., adolescen*, teen*, LMIC, middle income countr*) across the title, abstract, and subject fields, rather than formal controlled vocabulary (e.g., MeSH) headings. This approach was selected to maximize search sensitivity, given that concepts such as individual LMIC country names and youth-related terms are not consistently or fully captured by controlled vocabulary across the five databases searched.

### Eligibility criteria

Adolescence was defined according to the WHO classification as the period from 10 to 19 years of age (World Health Organization, 2024a). Studies were eligible for inclusion if they met the following criteria: (1) published in English between January 1, 2013 to July 31, 2024; (2) sample age range of study participants was 10 to 19 years old; (3) conducted in an LMIC, as defined by World Bank classifications (World Bank, 2022); (4) participants with subclinical anxiety or depression (i.e., symptoms of anxiety or depression), who did not meet the clinical criteria; (5) quasi-experimental and experimental designs (i.e., pre-post designs with a control or comparison group); (6) quantitative evaluations of the effectiveness of the intervention on depression or anxiety. Studies were excluded from this review if they were: (1) medication-only interventions; (2) interventions measured depression and anxiety with one overall instrument (i.e., with no separate scores for depression and anxiety); (3) study populations focused on extreme trauma experiences (e.g., abduction, rape).

### Data extraction

The following information was extracted from each study: (a) characteristics of studies (title, authors, year); (b) country; (c) characteristics of the intervention (theoretical foundation, dosage, format, setting, service provider); (d) study design (type of control, inclusion/exclusion criteria, blinding of assessors, fidelity assessment, measures, statistical analysis, missing data); (e) characteristics of the sample (age range, sample size, mean, standard deviation), and (f) outcome measure of depression and anxiety. One author (S. Y.) conducted data extraction, which was independently verified by a second author (T. T.).

### Methodological quality and risk of bias assessments

The methodological quality of the studies was assessed by two independent reviewers using an adapted version of the Methodological Quality Rating Scale (MQRS) by Auslander et al. (2012) displayed in Table 2. The MQRS has been successfully used in prior systematic reviews (e.g., Cabassa and Hansen, 2007; Galvin and Byansi, 2020; Hawes et al., 2021). To adapt the MQRS for this area of study, the present study made the following modifications: (1) Statistical power was added as a criterion to determine whether the study had adequate sample size and statistical power; and (2) The scale item assessing whether multiple sites were used was deleted since it was not applicable to this area of study. The possible total scale scores for the adapted MQRS ranged from 0 (low) to 13 (high), with higher scores indicating higher methodological quality. A mean split was used to determine which studies had higher versus lower methodological quality.

**Table 2:** Methodological quality rating scale for depression and anxiety intervention studies.

Methodological criteria	Rating (points)
1. Study design	0 = Single Group pretest/posttest
	1 = Quasi-experiment (e.g., matched control group)
	2 = Randomization with control group
2. Theoretical foundation	0 = Treatment not theoretically based or not reported
	1 = Theoretical basis discussed or cited
3. Treatment integrity	0 = No standardization specified or cited
	1 = Intervention standardized by manual procedures, specific training.
4. Measures	0 = Reliability and validity of measures not reported or inadequate
	1 = Reliability and validity of measures adequate
5. Length of follow-up	0 = No follow-up (pre and post-only)
	1 = Follow up was done (pre, post and follow-up)
6. Dosage	0 = Dosage was relevant but not discussed
	1 = Dosage was discussed or was not relevant to the intervention
7. Dropouts/attrition	0 = No discussion of dropouts
	1 = Dropouts enumerated and/or discussed
8. Blinding	0 = Follow-up or post-test conducted by non-blind or by an unspecified person
	1 = Follow-up or post-test by person blind to participants’ treatment condition
9. Analysis	0 = No statistical analysis conducted
	1 = Appropriate statistical analyses (between group differences, control for baseline differences)
10. Statistical power	0 = Clearly inadequate power due to small sample size or dropout or non-normal distribution
	1 = Adequate power with adequate sample size
11. Multi-site	0 = Single site study
	1 = Conducted at two or more sites
12. Generalizability	0 = No discussion of generalizability of findings
	1 = Discussion of generalizability of results (e.g., sample characteristics, site, treatment)

Scores can range from “0” (low) to “13” (high).

To assess bias of included studies, the Cochrane Risk of Bias tool version 2 (RoB 2.0; Sterne et al., 2019) was used for RCTs and the ROBINS-I (Sterne et al., 2016) for studies with quasi-experimental designs. RCTs were classified as ‘low risk,’ ‘some concerns,’ or ‘high risk’ of bias, and quasi-experimental studies were classified as ‘low,’ ‘moderate,’ ‘serious,’ or ‘critical’ risk of bias. All discrepancies were discussed between the two independent raters (YSY and TT) and resolved.

### Quality of evidence assessment

The quality of evidence was assessed using the Grading of Recommendations Assessment, Development and Evaluation (GRADE) approach (Guyatt et al., 2008). The GRADE assessment was used to compare evidence on intervention settings and deliverer types. Interventions conducted within school settings were categorized as school-based interventions, and non-school-based interventions that included hospitals, community-based organizations, and online platforms. A specialist was defined as a service provider who was a licensed clinical mental health professional and/or who received professional training at the master’s or doctoral level in mental health areas. A non-specialist was defined as a service provider who was trained to deliver the intervention but had no formal professional or paraprofessional certificate or post-secondary educational degree related to mental health (World Health Organization, 2007).

GRADE assessment included four dimensions: risk of bias, inconsistency, imprecision, and indirectness. Risk of bias is based on ratings from RoB 2.0 or ROBINS-I. Inconsistency reflects the median and range of Cohen’s d within each subgroup and whether the confidence intervals of the smallest- and largest-effect studies overlap (Higgins et al., 2024, Cochrane Handbook Chapter 12.2.1.1). Imprecision reflects the proportion of studies rated as adequately powered on the Methodological Quality Rating Scale (MQRS; Auslander et al., 2012) and total sample size contributing to each subgroup. Indirectness reflects whether the interventions align with the review’s target. Studies were classified as ‘high’, ‘moderate’, ‘low’ and ‘very low’ in quality of evidence. All discrepancies were discussed between the two independent raters (YSY and TT) and resolved.

### Data synthesis

Included studies used various measurements on anxiety and depression, and also various clinical models. Due to the heterogeneity of the included study methodologies and intervention types a formal meta-analysis was not conducted. However, standardized effect sizes (Cohen’s *d*) were calculated for each study from the reported means, standard deviations, and sample sizes (displayed in Appendix Tables 1, 2), and a narrative synthesis of all included studies was conducted.

## Results

### Selection and inclusion of studies

The initial database search identified 1,710 articles. After deleting 368 duplicates, 1,342 articles were eligible for abstract screening. Two researchers independently screened the titles and abstracts according to predefined inclusion and exclusion criteria, resulting in 57 articles for full-text review. During the full-text review process, an additional 35 studies were excluded, so 22 articles were included in the systematic review (see Figure 1. PRISMA Flow Diagram). Inter-rater agreement was assessed using Cohen’s kappa (κ = 0.85). Discrepancies were resolved through discussion to reach consensus.

**Figure 1:**
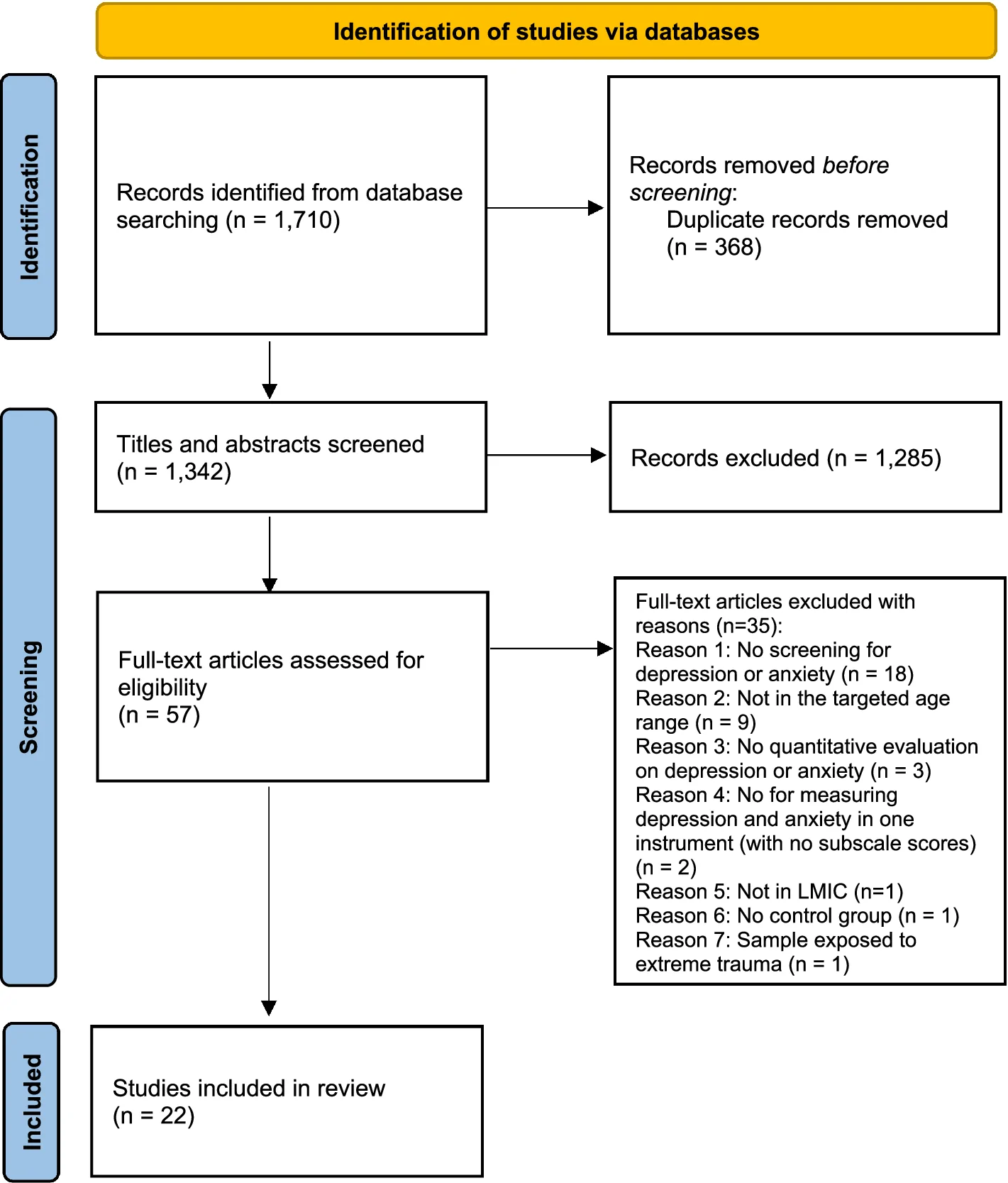
PRISMA flow diagram. The note said that this figure is adapted from the PRISMA 2020 flow diagram template (Page et al., 2021), licensed under CC BY 4.0. To view a copy of this license, visit https://creativecommons.org/licenses/by/4.0/.

### Characteristics of included studies

Table 3 summarizes the main characteristics of the included studies. We identified 22 studies with sample sizes ranging from 28 to 440 participants per study, implemented across 11 countries. Intervention dosage varied, with four studies having less than 4 sessions, 12 having 4 to 12 sessions, and 6 studies having over eight sessions. As for outcomes, seven studies measured depression, 10 studies measured anxiety, and 5 studies measured both. Among those measuring depression, the most common instrument used (*n* = 4) was an adapted version of the Patient Health Questionnaire (PHQ-9), a widely validated tool, while the remaining studies used different depression scales. For anxiety, there was greater variation; 6 studies assessed general anxiety symptoms but employed different instruments across all studies. Four studies focused on specific subtypes of anxiety such as social anxiety or test anxiety, each using distinct measures.

**Table 3:** Intervention characteristics summary (*N* = 22).

Authors (Years)	Country	Theoretical model	Age range	Treatment provider	Intervention setting	Dosage	Sample size (Intervention VS Control)	Depression measures	Anxiety measures
Alavi et al. (2013)	Iran	Cognitive Behavior Therapy	12–18 Years	Specialists	Non-school	> 8 sessions	15 VS 15	BDI	NA
Nasizadeh et al. (2015)	Iran	Dialectical Behavior Therapy	No information	Specialists	School	4 ~ 8 sessions	33 VS 33	NA	TAI
Shen et al. (2018)	China	Positive Psychology	13–15 Years	Nonspecialist	School	≤ 4 sessions	38 VS 37	NA	TAS-C
Saw et al. (2019)	Malaysia	Cognitive Behavior Therapy	16 Years	Specialists	School	4 ~ 8 sessions	10 VS 10	NA	HADS-Chinese Version
Moeini et al. (2019)	Iran	Cognitive Behavior Therapy	15–18 Years	Nonspecialist	Non-school	4 ~ 8 sessions	63 VS 64	RASD	NA
Kwok (2019)	Hong Kong, China	Positive Psychology	11–16 Years	Specialists	School	4 ~ 8 sessions	53 VS 53	CESD	NA
Osborn et al. (2020)	Kenya	Positive Psychology	12–19 Years	Nonspecialist	School	≤ 4 sessions	28 VS 23	PHQ-9	GAD-7
Amin et al. (2020)	Pakistan	Cognitive Behavior Therapy	13–16 Years	Specialists	School	4 ~ 8 sessions	38 VS 38	NA	LSA-CA
Osborn et al. (2021)	Kenya	Positive Psychology	13–18 Years	Nonspecialist	School	≤ 4 sessions	205 VS 208	PHQ-9	GAD-7
Jibunoh and Ani (2022)	Nigeria	Psychoeducation	13–16 Years	Specialists	School	≤ 4 sessions	20 VS 20	SMFQ	SCAS
Kwok et al. (2022)	Hong Kong, China	Positive Psychology	12–14 Years	Specialists	School	4 ~ 8 sessions	46 VS 46	NA	SCARED
Moloudi et al. (2022)	Iran	Cognitive Behavior Therapy	10–13 Years	Specialists	Non-school	> 8 sessions	14 VS 14	HADS	NA
Hamdani et al. (2022)	Pakistan	Early Adolescent Skills for Emotions	13–15 Years	Nonspecialist	School	4 ~ 8 sessions	28 VS 30	NA	HARS
Nazari et al. (2022)	Iran	Attention Control	15–17 Years	Nonspecialist	Non-school	3 weeks, every day	26 VS 26	PHQ-9	NA
Javadian and Sabet Eqlidi (2022)	Iran	Solution-focused Therapy	All 8th grade. No specific age information	Specialists	School	4 ~ 8 sessions	15 VS 15	NA	STAI
Bryant et al. (2022)	Jordan	Early Adolescent Skills for Emotions	10–14 Years	Nonspecialist	Non-school	4 ~ 8 sessions	175 VS 265	PHQ-Adolescent	NA
Ediz and Kavak Budak (2023)	Turkey	Psychoeducation	17–19 Years	Specialists	Non-school	4 ~ 8 sessions	50 VS 55	EPDS	BAI
Jordans et al. (2023)	Lebanon	Early Adolescent Skills for Emotions	10–14 Years	Nonspecialist	Non-school	4 ~ 8 sessions	80 VS 118	PHQ-Adolescent	NA
Sorsdahl et al. (2023)	South Africa	Cognitive Behavior Therapy	15–18 Years	Specialists	Non-school	4 ~ 8 sessions	60 VS 57	CESD	GAD-7
Soleimani Rad et al. (2023)	Iran	Cognitive Behavior Therapy	15–19 Years	Specialists	Non-school	> 8 sessions	18 VS 18	NA	SAS for Adolescents
Egbe et al. (2023)	Nigeria	Cognitive Behavior Therapy	11–18 Years	Specialists	School	> 8 sessions	61 VS 61	NA	LSAS-CA
Zemestani et al. (2024)	Iran	Transdiagnostic Treatment of Emotional Disorders	15–17 Years	Specialists	Non-school	> 8 sessions	28 VS 26	NA	Youth Anxiety Measure for DSM-5

Measures used in the included interventions to assess depression and/or anxiety symptoms are abbreviated in the table as follows: BDI, Beck Depression Inventory; RASD, Reynolds Adolescent Depression Scale – Second Edition; CES-D, Center for Epidemiologic Studies Depression Scale; PHQ-9, Patient Health Questionnaire–9; SMFQ, Short Mood and Feelings Questionnaire; HADS, Hospital Anxiety and Depression Scale; EPDS, Edinburgh Postnatal Depression Scale; TAI, Spielberger’s Test Anxiety Inventory; TAS-C, Test Anxiety Scale – Chinese Version; GAD-7, Generalized Anxiety Disorder–7; LSAS-CA, Liebowitz Social Anxiety Scale for Children and Adolescents; SCAS, Spence Children’s Anxiety Scale; SCARED, Screen for Child Anxiety Related Emotional Disorders; HARS, Hamilton Anxiety Rating Scale; STAI, State–Trait Anxiety Inventory; BAI, Beck Anxiety Inventory; SAS-A, Social Anxiety Scale for Adolescents. NA means Not Applicable.

The interventions were grounded in diverse theoretical models. The most common intervention theory employed was Cognitive Behavior Therapy (*n* = 8), five used positive psychology, three used the WHO-developed Early Adolescent Skills for Emotions (EASE), and six used other clinical models, including Dialectical Behavior Therapy, Transdiagnostic Treatment, Solution-focused Therapy, Psychoeducation, Acceptance Commitment Therapy, and Attention Control Theory. Interventions were implemented across different settings, with 12 studies conducted in schools and 10 in non-school environments such as hospitals, community-based NGOs, and online platforms. Characteristics of those delivering the intervention also varied, with 14 studies provided by specialists and eight by non-specialists. Geographically, as shown in Table 3, the studies were conducted across 10 different LMICs, with nearly half in the Middle East and North African region (8 from Iran, 1 from Lebanon, and 1 from Jordan) and three studies from China.

### Risk of bias

The overall risk of bias for each study is outlined in Tables 4, 5. Among the included studies, three are quasi-experimental studies, and 19 are randomized controlled trials (RCTs). Out of the 19 RCTs, six were rated as low risk, 12 as some concerns, and one as high risk. The main reasons for the “some concerns” ratings were bias in measurement of the outcome (4 studies did not use blinded outcome assessors) and bias in selection of the reported result (10 studies lacked a pre-registered protocol with a pre-specified data analysis plan). The one high-risk study had missing outcome data but did not report the steps taken to address it.

**Table 4:** Rob2 risk of bias ratings for RCTs (*N* = 19).

Study	Risk of bias domain					Overall
	D1	D2	D3	D4	D5	
Alavi et al. (2013)	+	+	+	−	−	−
Amin et al. (2020)	+	+	+	+	−	−
Bryant et al. (2022)	+	+	+	+	+	+
Ediz and Kavak Budak (2023)	+	+	−	+	−	−
Egbe et al. (2023)	+	+	+	−	−	−
Hamdani et al. (2022)	+	+	+	+	+	+
Jordans et al. (2023)	+	+	+	+	+	+
Kwok (2019)	+	−	+	+	−	−
Kwok et al. (2022)	+	+	+	+	−	−
Moeini et al. (2019)	+	+	−	+	+	−
Moloudi et al. (2022)	+	+	+	+	−	−
Nazari et al. (2022)	+	+	+	+	−	−
Osborn et al. (2020)	+	+	+	+	−	−
Osborn et al. (2021)	+	+	+	+	+	+
Saw et al. (2019)	+	+	+	−	−	−
Shen et al. (2018)	+	+	+	+	+	+
Soleimani Rad et al. (2023)	+	+	×	+	−	×
Sorsdahl et al. (2023)	+	+	+	+	+	+
Zemestani et al. (2024)	+	+	+	−	+	−

D1, bias arising from the randomization process; D2, bias due to deviations from intended interventions; D3, bias due to missing outcome data; D4, bias in measurement of the outcome; D5, bias in selection of the reported result. Judgment: 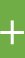, low risk; 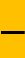, some concerns; 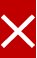, high risk.

**Table 5:** ROBINS -1 risk of bias ratings for non-randomized (quasi-experimental) studies (*N* = 3).

Study	Risk of bias domain						Overall
	D1	D2	D3	D4	D5	D6	
Javadian and Sabet Eqlidi (2022)	×	−	+	+	−	−	×
Jibunoh and Ani (2022)	×	+	+	+	+	−	×
Nasizadeh et al. (2015)	+	−	+	+	−	×	×

D1, bias due to confounding; D2, bias in classification of interventions; D3, bias in selection of participants into the study; D4, bias due to missing data; D5, bias in measurement of the outcome; D6, bias in selection of the reported result. Judgment: 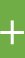, low risk; 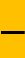, moderate risk; 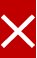, serious risk. RoB 2.0 and ROBINS-I use different domains and judgment scales and are therefore reported in separate tables.

All three quasi-experimental studies were rated at serious risk of bias. Two of the three showed major baseline differences in outcomes between the intervention and control groups, raising concerns about inadequately controlled confounding (Javadian and Sabet Eqlidi, 2022; Jibunoh and Ani, 2022). The remaining study showed serious risk of bias in the selection of the reported result (Nasizadeh et al., 2015).

### Methodological rigor

Table 6 displays the MQRS ratings for each study by item in the scale and the total scale scores. Analyses of the MQRS scores indicated that of a possible range of 0–13, the actual scores ranged from 5 to 12 with a median of 10 (*M* = 9.73, SD = 2.21). Using the mean split of the total scale scores, 15 of 22 studies showed higher methodological rigor (MQRS > 9.73), and 7 of 22 had lower rigor (MQRS ≤ 9.73), as shown in Figure 2. Results indicated that the methodological strengths across the studies included theoretically informed interventions, a discussion of the dosage, and adequate, reliable measures with reported high internal consistency. However, several methodological weaknesses were identified. First, 64% of studies (*n* = 14) lacked adequate statistical power, often due to small sample sizes and participant dropout. Second, 59% of studies (*n* = 13) lacked a follow-up phase to track whether intervention effects were maintained over time. Third, 36% of studies (*n* = 8) did not discuss the generalizability of their findings.

**Table 6:** MQRS rating table (*N* = 22).

No.	Study	Study design	Theoretical foundation	Treatment integrity	Measures	Follow-up length	Dosage	Dropout	Blinding	Analysis	Statistical power	Generalizability	Total scores
1	Alavi et al. (2013)	2	1	0	1	0	1	0	0	0	0	0	5
2	Nasizadeh et al. (2015)	1	1	1	1	0	1	0	0	1	0	0	7
3	Jibunoh and Ani (2022)	1	1	1	1	0	1	1	0	1	0	1	8
4	Javadian and Sabet Eqlidi (2022)	1	1	1	1	0	1	1	0	1	0	1	8
5	Osborn et al. (2020)	2	1	1	1	0	1	1	1	1	0	0	9
6	Ediz and Kavak Budak (2023)	2	1	1	1	0	1	1	1	0	1	0	9
7	Kwok (2019)	2	1	1	1	0	1	1	1	1	0	0	9
8	Kwok et al. (2022)	2	1	1	1	0	1	1	1	1	0	1	10
9	Moloudi et al. (2022)	2	1	1	1	0	1	1	1	1	0	1	10
10	Amin et al. (2020)	2	1	1	1	0	1	1	1	1	0	1	10
11	Osborn et al. (2021)	2	1	1	1	1	1	1	0	1	1	0	10
12	Saw et al. (2019)	2	1	1	1	1	1	1	0	1	0	1	10
13	Shen et al. (2018)	2	1	0	1	0	1	1	1	1	1	1	10
14	Zemestani et al. (2024)	2	1	1	1	1	1	1	0	1	0	1	10
15	Jordans et al. (2023)	2	1	1	1	1	1	1	1	1	0	0	10
16	Moeini et al. (2019)	2	1	1	1	0	1	1	1	1	1	1	11
17	Hamdani et al. (2022)	2	1	1	1	1	1	1	1	1	1	0	11
18	Nazari et al. (2022)	2	1	1	1	0	1	1	1	1	1	1	11
19	Sorsdahl et al. (2023)	2	1	1	1	1	1	1	1	1	0	1	11
20	Soleimani Rad et al. (2023)	2	1	1	1	1	1	1	1	1	0	1	11
21	Bryant et al. (2022)	2	1	1	1	1	1	1	1	1	1	1	12
22	Egbe et al. (2023)	2	1	1	1	1	1	1	1	1	1	1	12
Mean for each item		1.86	1	0.91	1	0.41	1	0.91	0.68	0.91	0.36	0.64	/
Total score for each item		41	22	20	22	9	22	20	15	20	8	14	/

All item scores range from 0 to 1, except for study design (0).

**Figure 2:**
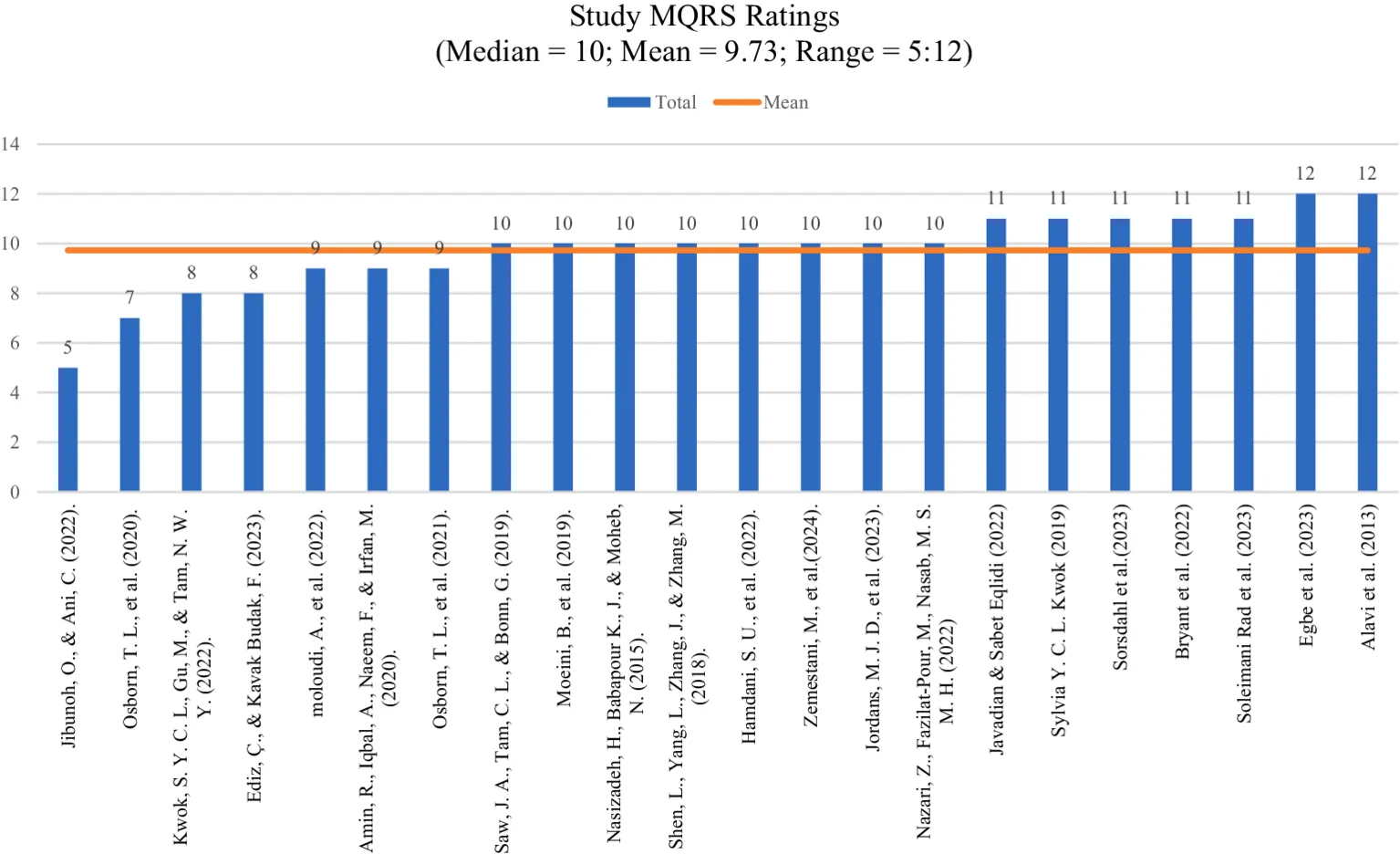
MQRS rating chart for all included studies (*N* = 22).

### Quality of evidence for school-based versus non-school-based interventions

As shown in Table 7, certainty of evidence for depression was rated very low regardless of setting. School-based interventions (*n* = 5, median *d* = 0.35, range 0.04–1.21) indicated risk of bias (one quasi-experimental study at serious risk), inconsistency (a moderate spread of effect sizes across studies), and imprecision (only 40% of studies adequately powered). Non-school-based interventions (*n* = 6, median *d* = 0.16, range 0.07–2.88) had no serious risk-of-bias concerns, but indicated concerns related to inconsistency, since the confidence intervals of the smallest- and largest-effect studies did not overlap, and for imprecision, as only half of the studies were adequately powered.

**Table 7:** Certainty of evidence (GRADE) for depression and anxiety by intervention setting (school-based vs. non-school-based).

GRADE evidence profile							
Outcome	Setting	No. of studies (design)	Risk of bias	Inconsistency	Indirectness	Imprecision	Certainty
Depression	School-based	5 (4 RCT, 1 Quasi)	Serious	Serious	Not serious	Serious	⊕◯◯◯ Very Low
	Non-school-based	6 (6 RCT)	Not serious	Very serious	Not serious	Serious	⊕◯◯◯ Very Low
Anxiety	School-based	10 (7 RCT, 3 Quasi)	Very serious	Very serious	Not serious	Serious	⊕◯◯◯ Very Low
	Non-school-based	6 (6 RCT)	Serious	Very serious	Not serious	Serious	⊕◯◯◯ Very Low

GRADE certainty ratings were derived using criteria appropriate for a systematic review without meta-analysis (SWiM; Campbell et al., 2020) rather than pooled effect estimates. Risk of bias reflects the proportion of contributing studies rated low/some concerns/high risk (RoB 2.0) or low/moderate/serious risk (ROBINS-I). Inconsistency reflects the median and range of Cohen’s d within each subgroup and whether the confidence intervals of the smallest- and largest-effect studies overlap (Higgins et al., 2024, Cochrane Handbook Chapter 12.2.1.1). Imprecision reflects the proportion of studies rated as adequately powered on the Methodological Quality Rating Scale (MQRS; Auslander et al., 2012) and total sample size contributing to each subgroup. Indirectness was rated not serious throughout, assuming the review’s PICO defines the intervention broadly.

For anxiety, both school-based (*n* = 10, median *d* = 0.60, range 0.21–2.62) and non-school-based (*n* = 6, median *d* = 1.48, range 0.45–2.87) interventions were also rated very low certainty. Both subgroups were downgraded for risk of bias (30–33% of studies at high or serious risk), inconsistency (non-overlapping confidence intervals between the smallest- and largest-effect studies), and imprecision (only 30–33% of studies adequately powered).

In sum, the very low certainty of evidence across all four subgroups indicates that the results cannot support a confident conclusion about whether school-based or non-school-based delivery is more effective for either outcome.

### Quality of evidence for specialist-delivered versus non-specialist-delivered interventions

As shown in Table 8, certainty of evidence for depression differed by deliverer. Specialist-delivered interventions (*n* = 5, median *d* = 1.01, range 0.04–2.88) indicated concerns for risk of bias (one quasi-experimental study at serious risk), inconsistency (a wide spread of effect sizes, with non-overlapping confidence intervals between the smallest- and largest-effect studies), and imprecision (only 20% of studies adequately powered), yielding very low certainty. Non-specialist-delivered interventions (*n* = 6, median *d* = 0.26, range 0.07–0.39) showed no serious concerns on any domain: risk of bias was low, effect sizes were narrow and consistent with overlapping confidence intervals, and 67% of studies were adequately powered, yielding high certainty.

**Table 8:** Certainty of evidence (GRADE) for depression and anxiety by intervention deliverer (specialist vs. non-specialist).

GRADE evidence profile							
Outcome	Deliverer	No. of studies (design)	Risk of bias	Inconsistency	Indirectness	Imprecision	Certainty
Depression	Specialist	5 (4 RCT, 1 Quasi)	Serious	Very serious	Not serious	Very serious	⊕◯◯◯ Very Low
	Non-specialist	6 (6 RCT)	Not serious	Not serious	Not serious	Not serious	⊕ ⊕ ⊕⊕ High
Anxiety	Specialist	12 (9 RCT, 3 Quasi)	Very serious	Very serious	Not serious	Very serious	⊕◯◯◯ Very Low
	Non-specialist	4 (4 RCT)	Not serious	Very serious	Not serious	Not serious	⊕◯◯◯ Low

GRADE certainty ratings were derived using criteria appropriate for a systematic review without meta-analysis (SWiM; Campbell et al., 2020) rather than pooled effect estimates. Risk of bias reflects the proportion of contributing studies rated low/some concerns/high risk (RoB 2.0) or low/moderate/serious risk (ROBINS-I). Inconsistency reflects the median and range of Cohen’s d within each subgroup and whether the confidence intervals of the smallest- and largest-effect studies overlap (Higgins et al., 2024, Cochrane Handbook Chapter 12.2.1.1). Imprecision reflects the proportion of studies rated as adequately powered on the Methodological Quality Rating Scaleand total sample size contributing to each subgroup. Indirectness was rated not serious throughout, assuming the review’s PICO defines the intervention broadly.

For anxiety, specialist-delivered interventions (*n* = 12, median *d* = 1.35, range 0.21–2.87) indicated risk of bias in terms of inconsistency and imprecision (only 17% of studies adequately powered), yielding very low certainty. Non-specialist-delivered interventions (*n* = 4, median *d* = 0.58, range 0.37–2.00) had a low risk of bias, and 75% of studies were adequately powered, but were downgraded for inconsistency because the confidence intervals of the smallest- and largest-effect studies did not overlap, yielding low certainty.

Overall, only non-specialist-delivered interventions for reducing depressive symptoms showed high certainty of evidence. All other comparisons (school versus non-school-based interventions, and specialist-delivered interventions) indicated low certainty for the evidence for their effectiveness.

## Discussion

The current systematic review examined the certainty of the evidence among interventions designed to reduce depression and anxiety among adolescents in LMICs, particularly comparing the effectiveness of school versus non-school-based interventions, as well as the training of the intervention deliverers (specialists versus non-specialists). To the best of our knowledge, this is the first systematic review to draw attention to the influence of contextual factors, specifically deliverer type and implementation setting, on the effectiveness of psychological interventions for adolescents in LMICs. Previous reviews have instead focused primarily on demographic differences and clinical models, and have also targeted promotions for the general population or subpopulations with clinically diagnosed disorders (Alozkan-Sever et al., 2023; Jokinen et al., 2026). This systematic review targets adolescents with subclinical symptoms of anxiety and depression, which is an important stage for preventing mental disorders. The search resulted in 22 trials with 2,336 participants across 11 countries. The findings suggest the potential effectiveness of non-specialist-delivered interventions against depression symptoms with high certainty, while the same cannot be concluded for anxiety symptoms. Similarly, the evidence was insufficient to determine whether school-based or non-school-based interventions were more effective for either outcome.

For school-based versus non-school-based delivery, certainty of evidence was very low across depression and anxiety outcomes. Current evidence therefore cannot determine which setting is more effective for depression or anxiety. This pattern is broadly consistent with Caldwell et al.'s (2019) large network meta-analysis, which similarly concluded that evidence for school-based preventive interventions was weak and not robust, and with Yin et al.'s (2025) LMIC-focused review, which likewise rated certainty of evidence as very low across outcomes and urged caution in interpreting its findings. In contrast, Werner-Seidler et al. (2021) drawing on a substantially larger and predominantly high-income-country sample, found small but statistically significant effects on both depressive and anxiety symptoms. Such inconsistency across the literature is often attributable to heterogeneity in inclusion criteria, variation in methodologies, and differences in geographical focus. For example, Werner-Seidler et al. (2021) restricted inclusion to randomized trials, whereas the present review also included quasi-experimental studies, all of which were rated at serious risk of bias. Additionally, within the present review specifically, effect sizes varied widely across studies within each setting (Cohen’s d ranging from 0.04 to 2.88 for depression and from 0.21 to 2.87 for anxiety), reflecting differences in sample size, outcome measures, and clinical modality. Included interventions ranged from brief, single-session digital programs to intensive, multi-session CBT protocols, and outcome measures varied across nearly every study. This degree of variation makes it difficult to isolate the effect of setting alone. It also calls for more rigorous, adequately powered trials using standardized outcome measures to assess how intervention effectiveness varies across implementation settings.

For provider type, findings point to the potential of non-specialist-delivered or task-shifting interventions in resource-limited settings, particularly for depression. Non-specialist-delivered interventions showed high certainty of benefit (*n* = 6), consistent with Karyotaki et al.'s (2022) meta-analysis of task-shared depression interventions in LMICs, and Ó hAnrachtaigh et al.’s (2024) review. With proper training and supervision, non-specialist providers can serve as an important workforce for expanding access to mental health services needed in resource-limited settings. For anxiety, however, the evidence base for non-specialist delivery was smaller. Only four interventions were delivered by non-specialists, compared to 12 delivered by specialists. This limited number of studies, alongside a wide spread of effect sizes, restricted the certainty of the evidence to low. Alozkan-Sever et al. (2023) reported that specialist-delivered interventions were more effective than non-specialist-delivered interventions for anxiety among LMIC youth meeting clinical or above-cutoff criteria. The present findings cannot confirm or refute this conclusion, as the small number of non-specialist-delivered anxiety studies in this review limits the certainty of any comparison. More research with a larger number of non-specialist-delivered anxiety interventions is needed to conduct a comparison on provider type. It is also worth noting that, unlike depression, the anxiety interventions included in this review targeted a range of distinct subtypes, including test anxiety, social anxiety, and generalized anxiety. Each subtype of anxiety potentially involves different change mechanisms and skill requirements. Future research could further examine whether specialist versus non-specialist delivery yields different outcomes across these anxiety subtypes, rather than treating anxiety as a single, undifferentiated target.

Heterogeneity is an important feature of the evidence base as a whole. The 22 included studies varied substantially in study design (19 RCTs and 3 quasi-experimental studies), theoretical models (CBT, positive psychology, psychoeducation, and others), dosage (ranging from a single session to more than eight sessions), and outcome measures (with anxiety assessed using at least six different instruments across studies). This heterogeneity limited the extent to which findings could be meaningfully pooled or compared and contributed directly to the inconsistency and imprecision concerns that reduced the certainty of the evidence in the GRADE comparisons. Similar heterogeneity has been noted as a consistent challenge in other LMIC-focused prevention and intervention literature (Caldwell et al., 2019; Yin et al., 2025).

Several included studies reported challenges related to participant attrition and reduced engagement throughout the intervention. Potential contributing factors discussed in the studies were transportation barriers, competing school or family responsibilities, stigma around seeking mental health support, or limited availability of trained providers (Jordans et al., 2023; Bryant et al., 2022; Osborn et al., 2021). These patterns align with broader evidence that access to mental health services in LMICs is shaped by structural factors, including workforce shortages, resource constraints, and social stigma, which can affect the feasibility and sustainability of interventions (Mascayano et al., 2015; Mohammadzadeh et al., 2020; Zemestani et al., 2024). Additionally, our findings also suggest cross-country variation in these challenges. For example, Iran may have a stronger mental health professional workforce and greater access to a scientific community, but many of these resources are more limited for implementation.

### Study limitations

The results of this study should be considered in light of several limitations. First, the sample size of this systematic review is relatively small, particularly when the analyses of effectiveness are conducted separately by each outcome (i.e., depression and anxiety). Second, subjective ratings were used to determine the methodological rigor of the included studies. Yet, potential biases were minimized by using a well-established measure and involving two independent raters who resolved any discrepancies through consensus. Third, our study excluded gray literature, which might have resulted in publication bias in this review. Fourth, not all countries or regions were equally represented in this systematic review [e.g., Middle East and SSA (*n* = 10), contrasting with South Asia (*n* = 2)]. These results suggest that while some countries like Iran may have a stronger mental health professional workforce and access to a scientific community, many LMICs face challenges to implementation with more limited resources. This uneven representation limits the generalizability and applicability of our findings across broader LMIC contexts. Fifthly, this review focused on populations with subclinical symptoms of anxiety and depression rather than clinically diagnosed disorders. Because symptoms are self-reported and subjective, individuals may perceive and report them differently depending on personal, social, and cultural context. This subjectivity may limit the comparability of findings across studies and should be considered when interpreting the results of this review. Last, this review only included publications in English, which may have biased our sample of studies included in the analyses.

### Future implications

Several research and practical implications can be drawn from the current findings. For example, future research could test interventions at multiple schools that may differ in sample characteristics or communities to demonstrate generalizability and ecological validity of the programs. Also, increasing sample sizes to address the lack of statistical power found among several studies in this review is warranted, as well as including longer follow-up phases to determine whether the intervention effects are sustained over time. Future research should also ensure that protocols are registered and that analysis plans are pre-registered to reduce the risk of selective reporting, as well as reporting effect sizes with sufficient precision to support meta-analysis where appropriate. Despite the use of reliable measures, the heterogeneity of measures, especially anxiety assessments, limits the ability to compare outcomes across different studies. Thus, this area of research would benefit from culturally validated and adapted measures that balance comparability across studies with sensitivity to how depression and anxiety symptoms may be experienced and expressed differently across social and cultural contexts in LMICs. Additionally, future research could further examine whether specialist versus non-specialist delivery yields different outcomes across anxiety subtypes, rather than treating anxiety as a single, undifferentiated construct.

## Conclusion

In summary, an important finding of this study indicates the potential effectiveness of training and supervising non-specialist providers consistent with task-shifting interventions in reducing adolescent depression symptoms, with high certainty of evidence supporting this approach. This review’s findings cannot determine whether school-based or non-school-based delivery is more effective for either outcome. Given the underdeveloped mental health workforce in LMICs, our findings suggest that future practice could prioritize training and supervising non-specialist providers, including teachers, lay counselors, and community health workers, to deliver evidence-based depression interventions, regardless of setting, in order to expand access and scale up mental health services for adolescents. Further rigorous research is needed to determine whether this task-shifting approach is similarly effective for reducing anxiety, and whether implementation settings (school or non-school) offer additional benefit.

## Data Availability

The original contributions presented in the study are included in the article/Supplementary material, further inquiries can be directed to the corresponding author.
