# Supplementary material for: Mental health interventions for adolescents with depression and anxiety in low- and middle-income countries: a systematic review of the evidence
Source: Front Psychol. 2026 Oct 19;17:1809434. doi: 10.3389/fpsyg.2026.1809434 (PMC13724535; doi:10.3389/fpsyg.2026.1809434)
Supplement: Supplementary file 1 [file Table_1.docx]

**Appendix 1.** Summary of Effect Sizes for Depression Outcomes (n = 11)

| **Study** | **N (Int./Ctrl.)** | **Intervention** | | | **Control** | | | **Cohen's d [95% CI]** | **Significance** |
| --- | --- | --- | --- | --- | --- | --- | --- | --- | --- |
|  |  | **Pre M (SD)** | **Post M (SD)** | **FU M (SD)** | **Pre M (SD)** | **Post M (SD)** | **FU M (SD)** |  |  |
| Alavi et al., 2013 | 15/15 | 30.58 (5.35) | 14.42 (3.89) | — | 27.78 (4.11) | 33.60 (8.56) | — | 2.88 [1.86, 3.91] | Significant |
| Bryant et al., 2022 | 175/265 | 15.06 (5.85) | 12.80 (4.90) | 12.42 (4.76)³ | 15.39 (4.57) | 12.37 (4.90) | 12.36 (4.74)³ | 0.09 [−0.10, 0.27] | Not significant |
| Ediz & Kavak Budak, 2023 | 50/55 | 15.01 (3.63) | 13.01 (3.84) | — | 15.92 (3.06) | 16.34 (2.73) | — | 1.01 [0.60, 1.41] | Significant |
| Hamdani et al., 2022 | 28/30 | 7.59 (3.91) | 3.71 (3.86) | — | 7.20 (5.06) | 5.64 (5.70) | — | 0.39 [−0.13, 0.91] | Not significant |
| Jibunoh & Ani, 2022 | 20/20 | 7.80 (5.04) | 9.20 (3.64) | — | 6.80 (4.48) | 9.05 (4.49) | — | 0.04 [−0.58, 0.66] | Not significant |
| Jordans et al., 2023 | 80/118 | 9.5 (7.16) | 8.3 (7.16) | 7.4 (8.05)³ | 9.6 (15.21) | 8.3 (15.21) | 8.3 (15.21)³ | 0.07 [−0.21, 0.35] | Not significant |
| Moeini et al., 2019 | 63/64 | 24.6 (11.7) | 18.5 (14.0) | 19.5 (10.9)³ | 22.3 (11.8) | 21.4 (15.6) | 22.5 (12.1)³ | 0.20 [−0.18, 0.58] | Not significant |
| Osborn et al., 2020 | 28/23 | 13.43 (4.14) | 10.21 (4.39) | — | 12.91 (3.52) | 12.52 (4.23) | — | 0.32 [−0.34, 0.99] | Significant |
| Osborn et al., 2021 | 205/208 | 12.9 (4.6) | 7.20 (4.92) | 8.01 (5.09)⁷ | 12.4 (4.8) | 8.34 (4.65) | 9.73 (5.30)⁷ | 0.35 [0.09, 0.60] | Significant |
| Saw et al., 2019 | 10/10 | 80.20 (4.83) | 68.90 (10.29) | 59.80 (14.94)¹ | 79.10 (3.67) | 77.80 (1.75) | 82.30 (4.30)¹ | 1.21 [0.25, 2.16] | Significant |
| Sorsdahl et al., 2023 | 60/57 | 14.47 (3.97) | 9.65 (4.92) | 10.21 (5.34)³ | 14.27 (3.98) | 10.20 (5.40) | 9.61 (5.91)³ | 0.11 [−0.32, 0.53] | Not significant |

*Note.* M = mean; SD = standard deviation; FU = follow-up; Int. = intervention group; Ctrl. = control group; CI = confidence interval. Positive Cohen's d values indicate greater symptom reduction in the intervention group relative to the control group. Significance was determined by whether the 95% confidence interval around the calculated Cohen's d excluded zero, rather than by the significance test reported in the original study, since included studies used different statistical methods to assess significance.

¹1-month follow-up. ³3-month follow-up. ⁷7-month follow-up.

**Appendix 2.** Summary of Effect Sizes for Anxiety Outcomes (n = 16)

| **Study** | **N (Int./Ctrl.)** | **Intervention** | | | **Control** | | | **Cohen's d [95% CI]** | **Significance** |
| --- | --- | --- | --- | --- | --- | --- | --- | --- | --- |
|  |  | **Pre M (SD)** | **Post M (SD)** | **FU M (SD)** | **Pre M (SD)** | **Post M (SD)** | **FU M (SD)** |  |  |
| Amin et al., 2020 | 38/38 | 82.02 (6.93) | 58.47 (10.72) | — | 84.87 (7.85) | 83.97 (8.60) | — | 2.62 [2.01, 3.24] | Significant |
| Ediz & Kavak Budak, 2023 | 50/55 | 47.30 (12.44) | 30.52 (6.46) | — | 46.18 (8.76) | 48.13 (14.16) | — | 1.58 [1.14, 2.01] | Significant |
| Egbe et al., 2023 | 61/61 | 57.23 (5.13) | 46.61 (5.23) | 28.91 (4.09)² | 56.67 (5.30) | 50.14 (5.62) | 33.66 (4.11)² | 0.65 [0.29, 1.01] | Significant |
| Javadian & Sabet Eqlidi, 2022 | 15/15 | 27.87 (5.22) | 19.81 (3.19) | — | 31.46 (4.34) | 29.10 (4.47) | — | 2.39 [1.46, 3.33] | Significant |
| Jibunoh & Ani, 2022 | 20/20 | 59.90 (4.78) | 56.00 (16.81) | — | 59.16 (5.58) | 48.95 (15.88) | — | 0.43 [−0.20, 1.06] | Not significant |
| Kwok, 2019 | 53/53 | 11.79 (3.95) | 9.01 (4.12) | — | 11.33 (3.92) | 9.88 (4.18) | — | 0.21 [−0.17, 0.59] | Not significant |
| Kwok et al., 2022 | 46/46 | 10.22 (3.69) | 6.76 (4.07) | — | 9.76 (3.92) | 8.54 (3.51) | — | 0.47 [0.05, 0.88] | Significant |
| Moloudi et al., 2022 | 14/14 | 81.67 (27.09) | 52.48 (15.54) | — | 81.34 (26.71) | 82.34 (26.48) | — | 1.38 [0.55, 2.20] | Significant |
| Nasizadeh et al., 2015 | 33/33 | 46.09 (9.82) | 31.79 (11.38) | — | 47.21 (10.00) | 46.61 (11.05) | — | 1.32 [0.79, 1.85] | Significant |
| Nazari et al., 2022 | 26/26 | 56.09 (4.70) | 48.33 (6.01) | — | 56.30 (5.68) | 52.55 (7.76) | — | 0.61 [0.02, 1.20] | Significant |
| Osborn et al., 2020 | 28/23 | 13.07 (4.05) | 9.29 (3.67) | — | 12.83 (2.33) | 11.65 (3.41) | — | 0.54 [−0.20, 1.29] | Not significant |
| Osborn et al., 2021 | 205/208 | 13.33 (3.37) | 6.95 (4.79) | 7.28 (4.89)⁷ | 13.25 (3.44) | 8.34 (4.26) | 9.07 (4.86)⁷ | 0.37 [0.11, 0.63] | Significant |
| Shen et al., 2018 | 38/37 | 24.05 (3.65) | 16.82 (3.68) | — | 23.73 (3.15) | 23.70 (3.17) | — | 2.00 [1.45, 2.56] | Significant |
| Soleimani Rad et al., 2023 | 18/18 | 48.17 (3.87) | 39.40 (4.04) | 40.61 (2.75)³ | 49.47 (5.71) | 51.31 (4.26) | 50.07 (4.14)³ | 2.87 [1.94, 3.80] | Significant |
| Sorsdahl et al., 2023 | 60/57 | 9.17 (4.75) | 5.63 (3.92) | 6.48 (4.56)³ | 9.04 (4.96) | 7.67 (4.99) | 6.77 (5.14)³ | 0.45 [0.04, 0.86] | Significant |
| Zemestani et al., 2024 | 28/26 | 101.51 (13.50) | 69.66 (14.69) | 73.57 (9.29)¹ | 98.66 (9.21) | 99.07 (9.98) | 99.19 (10.32)¹ | 2.31 [1.63, 2.98] | Significant |

*Note.* M = mean; SD = standard deviation; FU = follow-up; Int. = intervention group; Ctrl. = control group; CI = confidence interval. Positive Cohen's d values indicate greater symptom reduction in the intervention group relative to the control group. Significance was determined by whether the 95% confidence interval around the calculated Cohen's d excluded zero, rather than by the significance test reported in the original study, since included studies used different statistical methods to assess significance.

¹1-month follow-up. ²2-month follow-up. ³3-month follow-up. ⁷7-month follow-up.
